# Testing the snake-detection hypothesis: larger early posterior negativity in humans to pictures of snakes than to pictures of other reptiles, spiders and slugs

**DOI:** 10.3389/fnhum.2014.00691

**Published:** 2014-09-04

**Authors:** Jan W. Van Strien, Ingmar H. A. Franken, Jorg Huijding

**Affiliations:** Institute of Psychology, Erasmus University RotterdamRotterdam, Netherlands

**Keywords:** early posterior negativity (EPN), snake fear, spider fear, evolution, snake detection hypothesis

## Abstract

According to the snake detection hypothesis (Isbell, 2006), fear specifically of snakes may have pushed evolutionary changes in the primate visual system allowing pre-attentional visual detection of fearful stimuli. A previous study demonstrated that snake pictures, when compared to spiders or bird pictures, draw more early attention as reflected by larger early posterior negativity (EPN). Here we report two studies that further tested the snake detection hypothesis. In Study 1, we tested whether the enlarged EPN is specific for snakes or also generalizes to other reptiles. Twenty-four healthy, non-phobic women watched the random rapid serial presentation of snake, crocodile, and turtle pictures. The EPN was scored as the mean activity at occipital electrodes (PO3, O1, Oz, PO4, O2) in the 225–300 ms time window after picture onset. The EPN was significantly larger for snake pictures than for pictures of the other reptiles. In Study 2, we tested whether disgust plays a role in the modulation of the EPN and whether preferential processing of snakes also can be found in men. 12 men and 12 women watched snake, spider, and slug pictures. Both men and women exhibited the largest EPN amplitudes to snake pictures, intermediate amplitudes to spider pictures and the smallest amplitudes to slug pictures. Disgust ratings were not associated with EPN amplitudes. The results replicate previous findings and suggest that ancestral priorities modulate the early capture of visual attention.

## Introduction

As snakes were probably the first predators of primates, snakes will phylogenetically be more fear-relevant to humans than other reptiles. According to the snake-detection hypothesis (Isbell, 2006, 2009), snakes may have been important agents of evolutionary changes in the primate visual system allowing rapid visual detection of fearful stimuli. It is well established that in humans the visual detection of snakes is faster than of other, less life-threatening stimuli (Öhman and Mineka, 2001; Öhman et al., 2001). An evolved and specialized visual monitoring system for the detection of animals posing deadly threat would be highly adaptive from an evolutionary perspective. Such a fear module is activated automatically by fear-relevant stimuli, and is largely independent of conscious cognition.

Recently, two studies have provided direct electrophysiological evidence for the snake-detection hypothesis in primates and humans (Van Le et al., 2013; Van Strien et al., 2014). Van Le et al. (2013) measured neuronal responses in the medial and dorsolateral pulvinar of macaque monkeys. These laboratory animals had no chance to encounter snakes before the experiment. The study demonstrated the existence of pulvinar neurons that respond selectively to visual images of snakes. These neurons responded faster and stronger to snakes stimuli than to (angry) monkey faces, monkey hands, or geometrical shapes. The authors note that the pulvinar is part of a fast visual information processing pathway from the retina and superior colliculus via the pulvinar to the amygdala, allowing the rapid automatic visual detection of fear-related stimuli (see also, Morris et al., 1999). Mineka and Öhman (2002) regard the amygdala as the key structure that is dedicated to the evolved fear module. Through its subcortical projections, the amygdala modulates autonomic and behavioral fear reactions, In addition, the amygdala modulates cortical activity through direct connections to prefrontal and cortical visual areas, permitting enhanced processing of emotional stimuli (e.g., Dolan, 2002; Tamietto and de Gelder, 2010). In support of an evolved fear module, an fMRI study with non-phobic participants demonstrated that the amygdala responds to threatening animals such as snakes and spiders but not to threatening objects (i.e., weapons) with comparable valence and arousal levels (Yang et al., 2012).

According to the snake detection hypothesis, snakes draw more early attention than other threatening animals including spiders, but the existing evidence is sparse. In fMRI studies, the explicit hypothesis of larger amygdala activation in response to snakes compared to spiders has not been tested with non-phobic participants. Dilger et al. (2003) found higher left amygdala activation for spiders than for snakes in spider phobics but not in controls. In a recent study, Schaefer et al. (2014) examined the neurobiological correlates of normal and phobic fear for snakes. Both snake phobic and non-phobic participants exhibited larger amygdala activation to video clips of attacking snakes compared to video clips of fishes, but the activation to snakes was larger in phobics than in controls. Interestingly, a more strongly activated and more extensive network (including parietal, motor, and orbitofrontal areas) was found for phobic fear compared to normative fear. This suggests that in snake phobic individuals not only the fear module is triggered by snake stimuli, but also a fight-or-flight response, and possibly also attempts to down-regulate the fear response.

In a previous study (Van Strien et al., 2014), we tested the snake detection hypothesis in non-phobic women using event-related potentials (ERPs). In that study, participants watched the random rapid serial visual presentation (RSVP) of 600 snake pictures, 600 spider pictures, and 600 bird pictures at a rate of three pictures per second. The RSVP procedure allows the rapid changing of affective quality of the stimuli and is thus appropriate to study fast early processing of these stimuli. This makes good evolutionary sense as in the natural environment the fast discrimination of potential threat has a clear survival advantage (Junghöfer et al., 2001). In addition, the large number of trials per participant results in clean (i.e., noise free) ERPs for each condition. We measured the ERP component peaking around 225–300 ms after stimulus onset at lateral occipital sites, the so-called Early Posterior Negativity (EPN).

The EPN is supposedly generated by a parieto-occipital network (Junghöfer et al., 2001) and is thought to result from automatic selective visual attention to emotional stimuli, which facilitates visual encoding (Schupp et al., 2003). Emotional stimuli may evoke priority processing because of their intrinsic affective significance, with the amygdala modulating activity in the visual cortex (Schupp et al., 2003; Vuilleumier, 2005; Pourtois et al., 2013). ERP studies have consistently found augmented EPN amplitudes in response to emotionally significant compared to neutral stimuli (e.g., Schupp et al., 2004, 2006b; Foti et al., 2009; Flaisch et al., 2011; Leite et al., 2012; Calvo and Beltrán, 2013). The augmented EPN in response to emotional stimuli is not altered by habituation (Schupp et al., 2006a; Van Strien et al., 2009). In their review of ERP findings on affective picture processing, Olofsson et al. (2008) conclude that the main theoretical interpretation of the EPN is that it indexes “natural selective attention”. This attentional process is associated with the functioning of motivational systems of approach and avoidance and is elicited particularly by stimuli of evolutionary significance (Schupp et al., 2006a). In line with previous research (e.g., Foti et al., 2009; Van Strien et al., 2009), we interpret larger EPN amplitudes in response to emotionally or evolutionary significant compared to neutral stimuli as reflecting larger automatic early attention capture. Direct evidence for a link between larger EPN amplitudes and allocation of early attention has been provided by a study employing the face-in-the-crowd task and event-related potentials to examine attention shifts in threat detection (Feldmann-Wüstefeld et al., 2011). These authors found a threat advantage in their behavioral results, while they also found a larger EPN for angry faces compared to happy faces.

In our previous ERP study (Van Strien et al., 2014), we found that the EPN amplitude was largest for snake pictures, intermediate for spider pictures and smallest for bird pictures. On fear questionnaires, the participants indicated that they had less fear of birds than of either spiders or snakes. There was no difference between self-reported fear of spiders and fear of snakes. Interestingly, the fear of spiders correlated with the EPN amplitude for spider pictures (i.e., more self-reported spider fear was associated with enhanced EPN activity), while fear of snakes was not correlated with the EPN amplitude for snake pictures. A possible explanation for this lack of association may be that fear of snake is less reliably reported by Northwestern Europeans and is most probably based on an imaginary encounter with a snake. Taken together, the results of that study suggest that ancestral priorities modulate the early capture of visual attention and that this attention appears to be innate for snake stimuli, and independent of reported fear.

Although the above studies provided some evidence for Isbell’s snake detection theory, several issues remain unanswered. First, is the preferential activity in early visual processes specific for snakes or is it a categorical reptile effect? In the above studies snakes were contrasted with monkey faces, geometrical shapes, spiders, or birds. It may be that preferential visual processing is not limited to snakes but is also found for other predatory reptiles (Sagan, 1977; Öhman and Mineka, 2003). Second, fear of snakes and spiders may be confounded with disgust. It may be that disgusting animals also enhance early visual processing. Third, as we only tested female participants in our previous study (Van Strien et al., 2014), the question remains whether the larger EPN amplitudes to snake pictures are also found in male participants. Here we report two ERP studies that address these issues.

In Study 1, we employed the RSVP of snake, crocodile, and turtle pictures. Given the fear-relevance of each animal category, it was expected that snake pictures would evoke the largest (i.e., most negative) EPN amplitudes, followed by crocodile pictures, and with the smallest amplitudes for turtle pictures.

In Study 2, we employed the RSVP of snake, spider and slug pictures. Besides snake and spider pictures, we selected slug pictures, because these pictures will be rated low on fear but high on disgust. In addition to the fear questionnaires, participants also completed disgust questionnaires regarding these animals. Fear of animals may be driven by fear of contamination. Matchett and Davey (1991) proposed that many animal fears function to prevent transmission of diseases and are mediated by a disgust response. According to Davey (1994), spider fear in particular is a consequence of the disgust-relevant status of spiders. This status resulted from the erroneous association of spiders with many devastating and incomprehensible diseases from the Middle Ages onward.

The neural substrates for fear and disgust overlap in the amygdala, and the occipital and prefrontal areas, while the substrate for disgust also specifically involves the insular cortex (Calder et al., 2001; Stark et al., 2007). The emotional modulation of visual ERP components by disgust is unclear. Krusemark and Li (2011) found diminished visual cortical electrical activity for disgust than for neutral and fearful stimuli. On the other hand Carretié et al. (2011) found larger P2-related cuneus activation for disgust then for neutral and fearful distractors in a digit categorization test. They interpreted the larger cuneus activation as reflecting enhanced automatic attention to disgusting stimuli.

Our hypothesis for the EPN is based on our previous research (Van Strien et al., 2014) and the notion that the EPN is modulated by phylogenetic fear relevance. Thus, we expected the largest (i.e., most negative) EPN amplitude in response to snake pictures, intermediate EPN amplitudes to spider pictures and the smallest EPN amplitudes to slug pictures. The influence of disgust on EPN amplitudes is explored by means of correlation analysis.

In Study 2, both men and women participated. Compared to men, women may be more prone to snake and spider phobias because of the potential survival cost to their children (Rakison, 2009) and hence women may exhibit higher attentional capture of snake and spider stimuli than men. It should be noted that Van Le et al. (2013) tested both a male and a female macaque monkey and allegedly found preferential activity of pulvinar neurons to snake images in both sexes. Therefore, we hypothesized that both men and women will show enhanced EPN amplitudes to snakes, but that women relative to men will show larger EPN enhancements.

## Study 1

### Method

#### Participants

Participants were 24 female university students with normal or corrected-to-normal vision. Two women were left-handed, the others were right-handed by self-report. Ages ranged from 18 to 25 years, with a mean age of 20.79 years. They participated for a monetary reward. The study was approved by the departmental ethics committee. All participants provided written informed consent.

#### Self-report measures

Prior to the experimental run, participants rated their fear of crocodiles, turtles, and snakes by means of questionnaires. These questionnaires were adapted versions of the Spider Phobia Questionnaire (SPQ; Klorman et al., 1974; Muris and Merckelbach, 1996). Each questionnaire contained 15 statements regarding fear of the specific species. With the statements rated on a 4-point scale, scores on each questionnaire could range from 0 (no fear) to 45 (very high fear).

#### Stimuli and procedure

Participants were seated in a dimly-lit, sound-attenuated room and watched the rapid serial presentation of 600 snake pictures, 600 crocodile pictures, and 600 turtle pictures. For each of these three stimulus categories, there were 10 different color pictures. Each picture showed a complete specimen against a natural background and was repeated 60 times. The pictures had a size of approximately 600 × 450 pixels, and were displayed against a medium gray background on a 20-inch PC monitor with a resolution of 1024 × 768 pixels. The pictures were viewed at a distance of approximately 135 cm, resulting in a visual angle of about 10.0° × 7.5°. The pictures were presented in random order, as a continuous RSVP series with a rate of three pictures per second (Schupp et al., 2006a; Van Strien et al., 2009, 2014). There were no fixation crosses or blank screens between stimuli. In this passive viewing task participants were instructed to attend to the pictures. Following the experimental run, participants performed a computerized Self-Assessment Manikin (SAM) questionnaire (Bradley and Lang, 1994) regarding valence and arousal ratings of all pictures on a 9-point scale.

#### EEG recording

EEG activity was recorded using a BioSemi Active-Two system from 32 pin type active Ag/AgCl electrodes mounted in an elastic cap according to the international 10–20 system. Electro-oculogram (EOG) activity was recorded from flat-type active electrodes placed above and beneath the left eye, and from electrodes at the outer canthus of each eye. An additional pin-type active electrode (common mode sense) and a pin-type passive electrode (driven right leg) were used to comprise a feedback loop for amplifier reference. The EEG and EOG data were digitized with a sampling rate of 512 Hz, a low-pass filter of 134 Hz, and 24-bit A/D conversion.

#### ERP data analysis

For the offline processing of the EEG data, Brain Vision Analyzer software 2.0 (Brainproducts, Gilching, Germany) was used. The EEG signals were referenced to an average reference, and phase-shift-free filtered with a band pass of 0.10–30 Hz (24 dB/Oct). Correction for ocular artifacts was done using the Gratton et al. (1983) algorithm. ERP epochs were extracted lasting from 50 ms before stimulus onset to 330 ms after stimulus onset. The ERP signals were defined relative to the mean amplitude of the prestimulus period. For each participant and each stimulus category (snake, crocodile, turtle), average ERPs were computed. All epochs with a baseline-to-peak amplitude difference larger than 100 μV or smaller than −100 μV on any channel were excluded from further analysis. The mean percentage of valid epochs at analysis-relevant electrodes was more than 99% for each condition. The EPN was scored at occipital electrodes (O1, O2, Oz, PO3, and PO4) and was measured as the mean activity in the 225–300 ms time window after stimulus onset (e.g., Van Strien et al., 2009).

#### Statistical analyses

For the fear, valence, and arousal ratings, repeated measures analyses of variance (ANOVAs) were employed with stimulus category (snakes, crocodiles, and turtles) as factor. For the EPN amplitudes a repeated-measures ANOVA was conducted, with stimulus category (snakes, crocodiles, and turtles) and electrode (O1, Oz, O2, PO3, PO4) as factors. When appropriate, Greenhouse-Geisser correction was applied. To explore the relationship between reported fear and the EPN amplitude, we calculated Pearson correlations between questionnaire scores and EPN amplitudes for snakes, crocodiles, and turtles, respectively. To reduce the total number of correlations, we employed one occipital cluster (comprising O1, O2, Oz, PO3, and PO4) for the EPN amplitude measure.

## Results and discussion

### Fear measures

Mean fear scores were 16.92 (*SD* = 10.05; range 2–45) on the snake questionnaire, 12.17 (*SD* = 6.06; range 3–28) on the crocodile questionnaire, and 5.46 (*SD* = 3.62; range 1–16) on the turtle questionnaire. The stimulus category effect was significant, *F*_(2,46)_ = 27.54, *ɛ* = 0.698, *p* < 0.001. Bonferroni corrected comparisons showed that all differences between the three categories were significant (all *p*-values ≤ 0.002). These fear-ratings confirm that snakes are considered by our participants as most fear-relevant, crocodiles as intermediate, and turtles as least fear-relevant.

### Valence and arousal ratings

The mean valence and arousal ratings for crocodile, turtle and snake pictures are given in Table 1. The main stimulus category effects were significant for both valence, *F*_(2,46)_ = 31.52, *ɛ* = 0.623, *p* < 0.001, and arousal, *F*_(2,46)_ = 30.63, *ɛ* = 0.645, *p* < 0.001. Bonferroni comparisons revealed that both crocodile and snake pictures were rated significantly lower for valence and higher for arousal when compared to turtle pictures (all *p*-values < 0.001). There were no differences in valence and arousal ratings between crocodile and snake pictures (both *p*-values > 0.33).

**Table 1:** **Study 1—participants’ mean arousal and valence ratings (and standard deviations)**.

Stimulus category	Valence (SD)	Arousal (SD)
Crocodile	3.46 (1.68)	4.92 (1.89)
Turtle	5.53 (1.37)	2.67 (1.57)
Snake	3.68 (1.75)	4.89 (1.93)

*Note. Valence and arousal ratings are based on a rating scale from 1 to 9*.

Thus, although snakes are considered as more fear-relevant than crocodiles, both categories are comparably rated on valence and arousal.

### EPN

**Figure 1:**
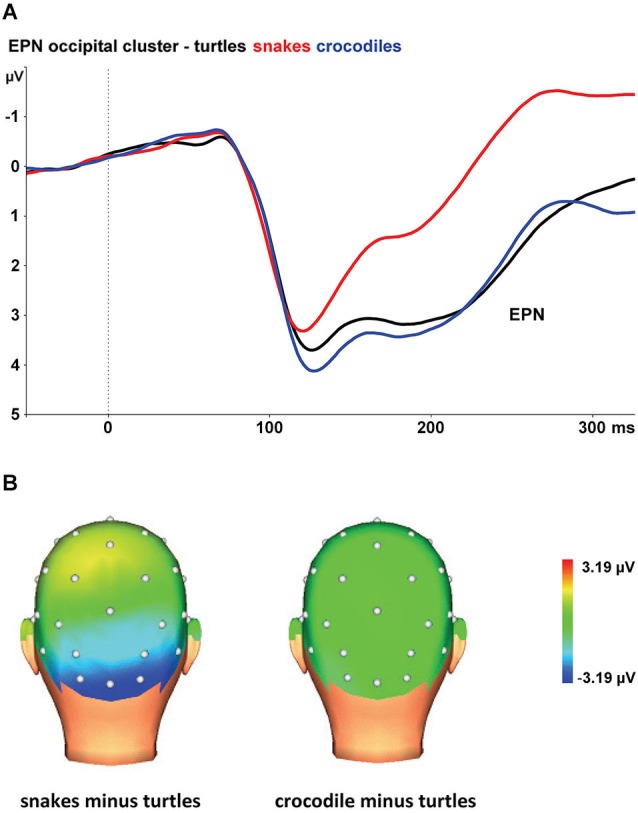
**(A)** The early posterior negativity (EPN) in response to snake (red lines), crocodile (blue lines), and turtle pictures (black lines) at the occipital cluster (Oz, O1/2, PO3/4). **(B)** Topographic maps of the differences in EPN mean amplitudes (225–300 ms) between snake vs. turtle pictures (left) and between crocodile vs. turtle pictures (right).

Figure 1A shows the grand average EPN potentials at the occipital cluster (O1, Oz, O2, PO3, PO4) for crocodile, turtle and snake pictures. Snake pictures yielded the most negative-going wave form, compared to both crocodile and turtle pictures. The ANOVA revealed a significant stimulus category effect, *F*_(2,46)_ = 126.17, *ɛ* = 0.697, *p* < 0.001. Bonferroni-corrected pairwise comparisons revealed that the EPN was significantly more negative for snake pictures than for both crocodile and turtle pictures (both *p*-value*s* < 0.001). For crocodile and turtle pictures, no significant difference in ERN amplitude emerged (*p* = 0.566).

The interaction of stimulus category and electrode was also significant, *F*_(8,184)_ = 15.06, *ɛ* = 0.446, *p* < 0.001. As can be seen in Figure 1B, the stimulus category effects are most pronounced at the occipital electrodes (O1, Oz, and O2), while the effects are present to a lesser degree at occipito-parietal electrodes (PO3 and PO4).

As expected, we found larger EPN amplitudes to snake pictures than to pictures of the other reptiles. Although crocodiles were rated as more fearful, unpleasant, and arousing than turtles, the EPN amplitudes were comparably low for crocodile and turtle pictures. Together, the results indicate that the EPN amplitude is not systematically related to reported fear and arousal, but is specifically elevated for snake pictures. These findings replicate previous research (Van Strien et al., 2014) that demonstrated preferential early capture of visual attention by snakes in particular.

### Correlation analyses

There were no robust correlations between the EPN cluster amplitude measures and the fear ratings for each stimulus category (−0.041 < *R* < 0.015). This is in accordance with previous research (Van Strien et al., 2014) that found no correlation between reported fear of snakes and the EPN amplitude.

## Study 2

### Method

#### Participants

Participants were 24 university students (12 men, 12 women) with normal or corrected-to-normal vision. Two participants (1 male, 1 female) were left-handed, the others were right-handed by self-report. Ages ranged from 20 to 27 years, with a mean age of 22.29 years. They participated for a monetary reward. The study was approved by the departmental ethics committee. All participants provided written informed consent.

#### Psychological measures

In addition to questionnaires regarding fear of snakes, slugs and spiders, the participants completed questionnaires regarding disgust for these animals prior to the experimental run. The disgust questionnaires were adapted versions of Armfield and Mattiske Disgust Scale (AMDS; Armfield and Mattiske, 1996). Each disgust scale contained eight statements regarding disgust for the specific species. With the statements rated on a 7-point scale, scores could range from 0 (no disgust) to 48 (very high disgust).

#### Stimuli and procedure

Participants watched the rapid serial presentation of 600 snake pictures, 600 spider pictures, and 600 slug pictures. Stimulus characteristics and procedures were further identical to those in Study 1.

#### EEG recording and data analysis

EEG recording, processing, and scoring were identical to Study 1. The mean percentage of valid epochs at analysis-relevant electrodes was more than 99% for each condition. For the fear, disgust, valence, and arousal ratings, mixed-design ANOVAs were employed with stimulus category (spiders, slugs, and snakes) as factor within subjects and sex as factor between subjects. For the EPN amplitudes a mixed-design ANOVA was conducted, with stimulus category and electrode as factors within subjects and sex as factor between subjects. When appropriate, Greenhouse-Geisser correction was applied. To explore the relationship between reported fear and disgust on the one hand, and the EPN amplitude on the other hand, we performed correlational analyses between questionnaire scores and EPN cluster (comprising O1, O2, Oz, PO3, and PO4) amplitudes for each stimulus category.

## Results and discussion

### Fear and disgust measures

Mean fear scores were 12.33 (*SD* = 7.15; range 2−30) on the spider questionnaire, 10.17 (*SD* = 5.25; range 4–27) on the slug questionnaire, and 9.33 (*SD* = 6.11; range 1–27) on the snake questionnaire. No significant main or interaction effects were found for these fear scores.

Mean disgust scores were 22.42 (*SD* = 9.59; range 3–44) on the spider questionnaire, 28.83 (*SD* = 10.48; range 0–42) on the slug questionnaire, and 20.79 (*SD* = 11.07; range 4–43) on the snake questionnaire. The stimulus category effect was significant, *F*_(2,44)_ = 6.44, *ɛ* = 0.903, *p* = 0.005. Bonferroni corrected comparisons showed that slugs were rated as more disgusting than snakes (*p* = 0.010) and spiders (*p* = 0.073). There was no difference between disgust ratings for spider and snake pictures (*p* = 1.00). Further, there was a main effect of sex, *F*_(1,22)_ = 4.36, *p* = 0.048, with women showing higher disgust scores (*M* = 27.17) than men (*M* = 20.86). Sex did not interact with the stimulus category effect.

Thus, we found no differences in fear ratings for snakes vs. spiders, which is consistent with our previous study (Van Strien et al., 2014). Contrary to our expectations, slugs were rated as fearful as snakes and spiders, but in accordance to our expectations slugs were rated as more disgusting than snakes and spiders. It may be that for slugs fear and disgust are confounded (i.e., slugs are fearful because they are disgusting), although we found no significant correlation between the participants’ fear and disgust ratings (*r* = 0.31, *p* = 0.146).

### Valence and arousal ratings

The mean valence and arousal ratings for spider, slug, and snake pictures are given in Table 2. The main stimulus category effects were significant for both valence, *F*_(2,44)_ = 7.58, *ɛ* = 0.913, *p* = 0.002, and arousal, *F*_(2,44)_ = 17.80, *ɛ* = 0.983, *p* < 0.001. Bonferroni comparisons revealed that spider pictures were rated significantly lower for valence when compared to slug (*p* = 0.027) and snake (*p* = 0.001) pictures. There was no difference in valence ratings between slug and snake pictures (*p* = 1.00). Spider (*p* < 0.001) and snake (*p* = 0.011) pictures were more arousing than slug pictures. Spider pictures tended to be more arousing than snake pictures (*p* = 0.058). For the valence ratings, we found a main effect of sex, *F*_(1,22)_ = 12.06, *p* = 0.002, with women showing lower valence ratings (*M* = 3.29; S*D* = 1.24) than men (*M* = 4.81; *SD* = 0.87). Sex did not interact with the stimulus category effects for the valence and arousal ratings. The lower valence and higher arousal ratings for spiders compared to snakes is consistent with our previous research. Although slugs were rated as more disgusting than snakes and spiders, they were rated as less arousing.

**Table 2:** **Study 2—participants’ mean arousal and valence ratings (and standard deviations)**.

Stimulus category	Valence (SD)	Arousal (SD)
Spider	3.37 (1.44)	4.49 (1.59)
Slug	4.32 (1.67)	2.56 (1.39)
Snake	4.47 (1.56)	3.69 (1.44)

*Note. Valence and arousal ratings are based on a rating scale from 1 to 9*.

### EPN

**Figure 2:**
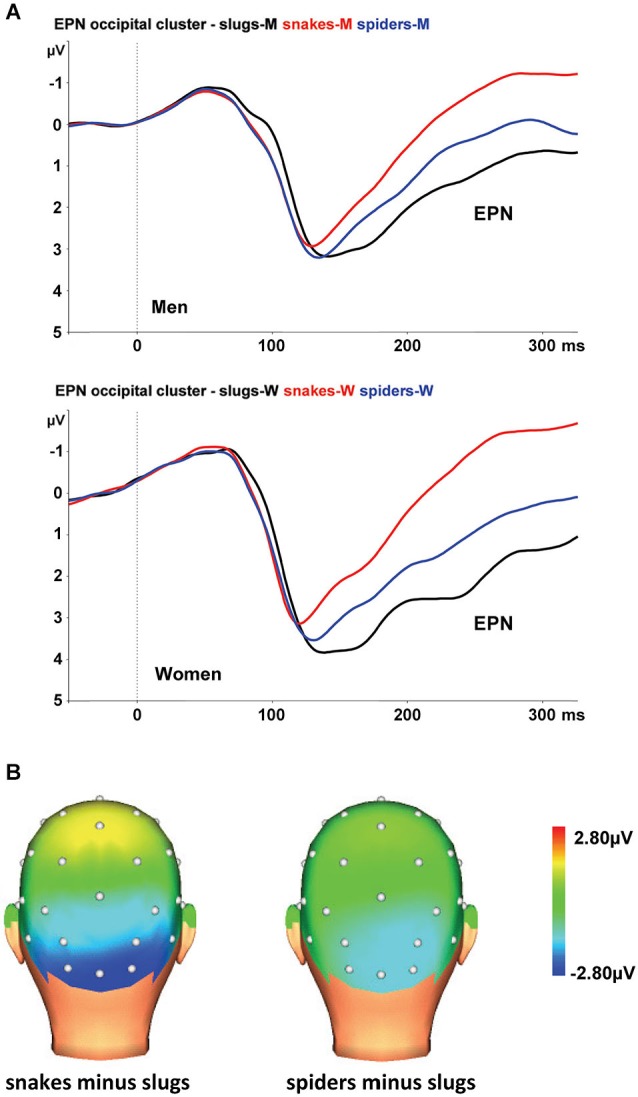
**(A)** The EPN in response to snake (red lines), spider (blue lines), and slug pictures (black lines) at the occipital cluster (Oz, O1/2, PO3/4) in men and women. **(B)** Topographic maps of the differences in EPN mean amplitudes (225–300 ms), across men and women, between snake vs. turtle pictures (left) and between crocodile vs. turtle pictures (right).

At the occipital cluster (O1, Oz, O2, PO3, PO4), snake pictures yielded the most negative-going wave form, followed by spider and slug pictures. The ANOVA revealed a significant stimulus category effect, *F*_(2,44)_ = 102.31, *ɛ* = 0.902, *p* < 0.001, that was qualified by a significant interaction of stimulus category and sex, *F*_(2,44)_ = 5.56, *ɛ* = 0.902, *p* = 0.009. This interaction is depicted in Figure 2A. Follow-up ANOVAs for men and women separately resulted in significant stimulus category effects in both groups (both *p*-values < 0.001). Within each group, all Bonferroni-corrected pairwise comparisons were significant (all *p*-values ≤ 0.004), indicating that both in men and women, the EPNs in response to the three stimulus categories differed significantly from each other. From Figure 2A, it can be seen that in women the EPN is more modulated than in men. To test the hypothesis that women relative to men will show larger EPN enhancements in response to snake pictures, we compared the snake minus slug EPN amplitudes in men and women. Compared to men, the EPN amplitude differences between the snake and slug categories was significantly larger in women (*p* = 0.005).

The interaction of stimulus category and electrode was also significant, *F*_(8,176)_ = 3.70, *ɛ* = 0.408, *p* = 0.013. As can be seen in Figure 2B, the stimulus category effects are most pronounced at the occipital electrodes (O1, Oz, and O2).

As expected, both males and females exhibited the largest EPN amplitudes to snake pictures, intermediate amplitudes to spider pictures, and the smallest amplitudes to slug pictures. The EPN modulations were most robust at occipital electrodes. These outcomes replicate our previous work (Van Strien et al., 2014) en demonstrate that the preferential early attentional capture by snake pictures is also found in men. Snake pictures modulated the EPN more substantially in women than in men, which we would tentatively suggest as support for the notion that women may be more prone to snake phobias because of potential survival costs to their children (Rakison, 2009).

Although snakes were not rated as more fearful and arousing than spiders, the EPN amplitudes were higher for snake than spider pictures. And although slugs were rated as more disgusting than snakes and spiders, the EPN amplitudes were lower for slug pictures than for snake and spider pictures. Together, the results show that the EPN amplitude is not systematically related to reported fear, arousal, or disgust but is specifically raised for snake pictures.

### Correlation analyses

There were no substantial correlations between the EPN cluster amplitude measures and fear ratings (0.039 < *r* < 0.050) or between the EPN cluster amplitude measure and disgust ratings (0.003 < *r* < 0.210). The absence of a correlation between spider fear ratings and EPN amplitudes is discordant with two previous studies. Van Strien et al. (2009) reported correlations from −0.37 to −0.47 between EPN amplitudes at lateral occipital sites and spider fear as measured on a 31-item yes/no questionnaire. Van Strien et al. (2014) found a correlation of −0.60 between the EPN at the lateral-occipital cluster of electrodes and spider fear as measured with the same questionnaire that was used in the present study. In the former studies, only women participated. Inspection of the present data revealed a higher, but nonsignificant, correlation in women (*r* = 0.21) than in men (*r* = 0.00). The numbers of male vs. female participants in the present study are too low to draw conclusions about these correlations, so the question to what degree the association between reported spider fear and EPN is existent in women, awaits further research.

## General discussion

In the present work, we examined by means of RSVP whether the preferential early attentional capture of snake stimuli as reflected in the EPN is limited to snakes or also extends to other reptiles (Study 1), whether the EPN is modulated by disgust, and whether the modulation of the EPN by phylogenetically fear-relevant snake stimuli is found both in women and men (Study 2).

We found that the EPN was larger to snake pictures than to pictures of other reptiles, and to pictures of spiders and slugs. The present studies clearly replicate the findings of our previous study in which we also found the largest EPN amplitudes in response to snake pictures (Van Strien et al., 2014). The differential EPN amplitudes at lateral occipital electrodes suggest that humans preferentially direct attention toward potentially threatening animate stimuli as part of motivational systems of approach and avoidance (Schupp et al., 2006a), with snakes capturing more early automatic attention than other reptiles, spiders, or slugs. As for the neurobiological substrate, the larger EPN amplitudes for snakes indicate increased source activity in the visual cortex (Schupp et al., 2003). Triggering the fear module may facilitate early visual processing, for instance by projections from the amygdala to visual cortical areas (Dolan, 2002; Tamietto and de Gelder, 2010). Interestingly, gene expressions appear to be associated with both enhanced amygdala activity during threat processing and enhanced EPN amplitudes, which suggests a link between amygdala activity and the EPN (Herrmann et al., 2007).

Study 1 demonstrates that amplified EPN amplitudes are specific in response to snake pictures and do not generalize to other reptiles. Although other reptiles may have been predators of primates as well, the EPN results suggest that the evolved fear detection module in the primate brain was predominantly designed to deal with snakes. It would be worthwhile to compare neural responses to snake and other reptile stimuli in macaque pulvinar neurons that respond selectively to snake pictures (cf., Van Le et al., 2013).

In Study 1, snakes were rated as more fearful than crocodiles, and crocodiles were rated as more fearful than turtles. In Study 2, snakes, spiders, and slugs were rated as equally fearful. EPN amplitudes were largest for snakes, intermediate for spiders and lowest for the other categories. So, no clear relation between self-reported fear for each stimulus category and corresponding grand-average EPN amplitude was found. A possible reason for the lack of association is that the EPN reflects early automatic attentional capture, which, especially for snakes, may be independent of consciously reported fear.

In Study 2, disgust ratings were, as expected, higher to slugs than to spiders or snakes. Women exhibited higher disgust scores than men for the animate stimuli, irrespective of stimulus category. This is in line with the fact that women typically report stronger feelings of disgust when compared to men (e.g., Rohrmann et al., 2008). As disgust ratings did not differ for spiders and snakes, disgust appears to be unrelated to the grand average EPN amplitudes for each stimulus category. Likewise, there was no correlation between participant’s disgust ratings for each category and the corresponding EPN amplitudes. In the present research with animate stimuli, disgust did not modulate the EPN. It should be noted that the mean disgust rating for slugs was relatively moderate (28.83 out of a maximum of 48) and that disgust as such can modulate the EPN. A recent study (Wheaton et al., 2013) found that, within a broad range of animate and inanimate stimuli, disgusting images (e.g., vomit, excrement, infections, contaminated food) evoked slightly higher EPN amplitudes than fearful images (e.g., angry faces, attacking dogs, weapons).

In Study 1, the snake and crocodile pictures were both rated as arousing and unpleasant, yet the EPN was much larger to snake than to crocodile pictures. In Study 2 and consistent with previous research (Van Strien et al., 2014), spiders were rated as more arousing and more unpleasant than snakes, yet the EPN was larger to snake than to spider pictures. Therefore, in this work with animate stimuli, conscious arousal and valence ratings appear not to be associated with the EPN. This is in agreement with the notion that the EPN reflects unintentional and automatic processing of emotional cues (Schupp et al., 2006a).

In Study 2, both men and women showed a clear enlargement of the EPN amplitude by snake pictures, with the EPN snake effect being larger in women than in men. This latter result makes evolutionary sense because women most probably had to protect themselves and their offspring against snakes (Rakison, 2009), resulting in better snake detection in women compared to men. Interestingly, a recent study demonstrated that women rather than men were distracted by peripherally presented snakes that were irrelevant for the task at hand (Öhman et al., 2012).

Other factors than predatory pressure may have influenced the larger EPN for snake pictures. One factor potentially modulating the EPN is picture complexity (i.e., simple figure-ground compositions vs. complex scenes; see Bradley et al., 2007; Nordström and Wiens, 2012). For all stimulus categories, we used simple figure-ground compositions that showed a single specimen in the foreground. Therefore it seems unlikely that picture complexity has confounded the present EPN results. A specific feature of snake stimuli, curvature in particular, may be absent in the other images and explain the enlarged EPN. It would be interesting to examine whether ambiguous stimuli that can be wrongly interpreted as snakes (such as a coiled rope or a garden hose) also evoke enlarged EPN. In our laboratory, research is on its way to address these issues. Meanwhile, an RSVP study that was published after we had conducted the present research, demonstrated larger EPN amplitudes to brightness equated grayscale pictures of snakes compared to spiders in a Japanese sample (He et al., 2014).

Inherent limitations of the ERP methodology, such as movements of current sources or differences between conditions in latency jitter (Otten and Rugg, 2005) might have affected the reported EPN amplitude differences. However, given the stable scalp distributions across conditions, the large number of epochs for the calculation of the ERPs, and in view of the abundant converging evidence of other neuroimaging studies, we think that the automatic allocation of early visual attention is the most straightforward explanation of the reported EPN amplitude enhancement for snake pictures.

Specific phobias can be classified into different categories, such as situational, animal and mutilation fears (Fredrikson et al., 1996) or phylogenetic (snakes, spiders) and ontogenetic (e.g., fear of flying) fears (Mühlberger et al., 2006). Different types of specific fear may be linked to different neurobiological substrates, with for instance an amygdala-based circuit for phylogenetic rather than for ontogenetic fears (Yang et al., 2012). Within the phylogenetic fear category, our results suggest that fear of snakes is more innate than fear of spiders. Therefore, the early automatic fear response will probably be more resistant to habituation in case of snake stimuli than in case of spiders or other phobic objects. Later attentional processes could be a better target of intervention, as in our former study with non-phobic participants (Van Strien et al., 2014), the non-speeded presentation of snake and spider pictures evoked comparable sustained attention (as measured with the late positive potential in the 500–900 ms time window after picture onset). It would be interesting to examine whether treatment for snake phobia differentially affects early vs. late attention-related ERPs (cf., Leutgeb et al., 2009).

In conclusion, as neither reported fear, disgust, valence, nor arousal seems to be systematically associated with the EPN amplitude, it seems that phylogenetically defined fear relevance modulates the EPN in the present studies. In accordance with our previous study, the present findings show that more early attention is allocated to snakes and spiders than to other reptiles or slugs. The automatic allocation of early visual attention is stronger for snakes than for spiders as snakes phylogenetically are more fear-relevant than spiders. The consistently larger EPN in the present and previous work for snake compared to other animate stimuli, including pictures of other reptiles, supports Isbell (2006, 2009) snake detection theory, which proposes that the neural defense circuitry in the primate brain was initially designed to deal with snakes. As a result, snakes still automatically draw attention in human beings. Our findings thus suggest that these ancestral priorities rather than consciously reported fear modulate the early capture of visual attention.

## Conflict of interest statement

The authors declare that the research was conducted in the absence of any commercial or financial relationships that could be construed as a potential conflict of interest.
